# Supplementary material for: Major antigenic differences in Aeromonas salmonicida isolates correlate with the emergence of a new strain causing furunculosis in Chilean salmon farms
Source: Front Cell Infect Microbiol. 2025 Feb 28;15:1508135. doi: 10.3389/fcimb.2025.1508135 (PMC11906462; doi:10.3389/fcimb.2025.1508135)

Table S1. Sequences of primers and probes for TaqMan assays used in this study.

| Marker | Forward primer | Reverse primer | Probe |
| --- | --- | --- | --- |
| *vapA* | ACTGTCTGTTACCCTGCCA | GCTACTTCACCCTGATTGG | FAM/ACATCAGCAGGCTTCAGAGTCACTG/IABkFQ |
| *fstA* | GTGACCGCCACCTATGAATC | CATCTTGTGCTGCTGGTCAT | FAM/TTCGGGGAAGAAAAACAGTG/IABkFQ* |

*IABkFQ is registered IDT quencher (https://www.idtdna.com/).

Table S2. Data of genome sequences used for phylogenomic analysis.

| Assembly Accession | Assembly BioSample Geographic location | Assembly BioSample Host | Strain_Name | Tree Host label |
| --- | --- | --- | --- | --- |
| GCA_006043955.1 | Unknown | Solea senegalensis | 23051 | Other fish |
| GCA_006044015.1 | Unknown | Solea senegalensis | 23053 | Other fish |
| GCA_006044075.1 | Unknown | Solea senegalensis | 23055 | Other fish |
| GCF_006044035.1 | Unknown | Solea senegalensis | 23056 | Other fish |
| GCF_001901975.1 | Canada | Salvelinus fontinalis | 01-B522 | Wild salmon |
| GCA_001902165.1 | Canada | Salmo salar | 09-0167 | Reared salmon |
| GCF_001901965.1 | Norway | Trout | 170-68 | Reared salmon |
| GCF_029069045.1 | Canada | Trout | 19-K304 | Reared salmon |
| GCF_029069085.1 | Canada | Trout | 19-K308 | Reared salmon |
| GCA_044881295.1 | Australia | Salmo salar | 20-3391-1 | Reared salmon |
| GCA_000786805.1 | Canada | Fish | 2004-05MF26 | Reared salmon |
| GCF_016811055.1 | Canada | Salmo salar | 2004-072 | Reared salmon |
| GCA_000786795.1 | Canada | Fish | 2009-144K3 | Reared salmon |
| GCF_001902045.1 | Canada | Salvelinus fontinalis | 2009-157_K5 | Wild salmon |
| GCF_001902055.1 | Canada | Salvelinus fontinalis | 2010-47_K18 | Wild salmon |
| GCA_000447435.2 | Argentina | Unknown | 34mel | Environmental |
| GCA_029153775.1 | USA | Oncorhynchus mykiss | AQ-22-VL-OH-WA-0003 | Reared salmon |
| GCA_029153815.1 | USA | Oncorhynchus mykiss | AQ-22-VL-OH-WA-0005 | Reared salmon |
| GCA_039659675.1 | USA | Oncorhynchus kisutch | AQ-22-VL-OH-WA-0021 | Reared salmon |
| GCA_032094615.1 | Canada | Anoplopoma fimbria | AQ-22-VL-ON-BC-0001 | Other fish |
| GCA_032094575.1 | Canada | Salmo salar | AQ-22-VL-ON-BC-0002 | Reared salmon |
| GCA_032094835.1 | Canada | Salmo salar | AQ-22-VL-ON-BC-0003 | Reared salmon |
| GCA_032094915.1 | Canada | Salmo salar | AQ-22-VL-ON-BC-0005 | Reared salmon |
| GCA_032094635.1 | Canada | Oncorhynchus tshawytscha | AQ-22-VL-ON-ON-0021 | Reared salmon |
| GCA_033880345.1 | Canada | Lepomis macrochirus | AQ-23-VL-ON-ON-0006 | Other fish |
| GCA_033880365.1 | Canada | Lepomis macrochirus | AQ-23-VL-ON-ON-0008 | Other fish |
| GCA_040501915.1 | Canada | Oncorhynchus tshawytscha | AQ-23-VL-ON-ON-0020 | Reared salmon |
| GCA_040502795.1 | Canada | Oncorhynchus kisutch | AQ-23-VL-ON-ON-0021 | Reared salmon |
| GCF_000315855.2 | South Korea | Carassius carassius | AS03 | Other fish |
| GCA_028356375.1 | China | Kareius bicoloratus | AS1 | Other fish |
| GCF_028358575.1 | China | Salmo salar | AS2 | Reared salmon |
| GCA_029541925.1 | China | Ctenopharyngodon idella | ASscau0313 | Other fish |
| GCF_001643305.1 | USA | Salmo salar | ATCC_33658 | Reared salmon |
|  | Chile | Salmo salar | AY-2485 | Reared salmon |
| GCF_016918885.1 | France | Salmo salar | BBCC2887 | Reared salmon |
| GCF_028356175.1 | China | Coregonus peled | BG | Other fish |
| GCA_014872735.1 | South Korea | Sebastes schlegeli | BR19001YR | Other fish |
| GCF_006246135.1 | Unknown | Fish | CIP_103210 | Reared salmon |
| GCF_006246315.1 | Unknown | Oncorhynchus mykiss | CIP_104001 | Reared salmon |
| GCF_006246305.1 | Unknown | Fish | CIP_104757 | Reared salmon |
| GCA_041861905.1 | Australia | Carp | F4 | Other fish |
| GCF_046105025.1 | China | Sturgeon | GMT3 | Other fish |
| GCF_001643275.2 | USA | Salmo salar | J223 | Reared salmon |
| GCF_027947655.1 | Canada | Fish | J225 | Reared salmon |
| GCF_027947675.1 | Canada | Fish | J228 | Reared salmon |
| GCA_009858115.1 | Canada | Sablefish | J409 | Other fish |
| GCA_009858135.1 | Canada | Sablefish | J410 | Other fish |
| GCA_012933685.1 | Canada | Sablefish | J411 | Other fish |
| GCF_018274765.1 | Chile | Salmo salar | JAOP-5 | Reared salmon |
| GCF_001901985.1 | Switzerland | Salvelinus alpinus | JF2267 | Wild salmon |
| GCA_001902065.1 | Norway | Salmo salar | JF2506 | Reared salmon |
| GCA_001902105.1 | United Kingdom | Salmo salar | JF2507 | Reared salmon |
| GCF_000931985.2 | Switzerland | Trout | JF3224 | Reared salmon |
| GCF_001902125.1 | Norway | Scophthalmus maximus | JF3517 | Other fish |
| GCF_001597895.1 | Switzerland | Salvelinus alpinus | JF3791 | Wild salmon |
| GCF_001466445.1 | Austria | Salvelinus alpinus | JF4097 | Wild salmon |
| GCA_029854275.1 | China | Fish | JNG | Reared salmon |
| GCA_033691215.1 | Peru | Oncorhynchus mykiss | JNO-10 | Reared salmon |
| GCA_033691235.1 | Peru | Oncorhynchus mykiss | JNO-11 | Reared salmon |
| GCA_033691055.1 | Peru | Oncorhynchus mykiss | JNO-12 | Reared salmon |
| GCA_033691155.1 | Peru | Oncorhynchus mykiss | JNO-6 | Reared salmon |
| GCA_033691175.1 | Peru | Oncorhynchus mykiss | JNO-7 | Reared salmon |
| GCF_004151085.1 | Canada | Salvelinus fontinalis | m11743-09 | Wild salmon |
| GCA_002110585.1 | Canada | Salvelinus fontinalis | M15879-11 | Wild salmon |
| GCA_005476635.1 | Canada | Salvelinus fontinalis | M16474-11 | Wild salmon |
| GCA_002883135.1 | Canada | Cyclopterus lumpus | M18076-11 | Other fish |
| GCA_002811185.1 | Canada | Salvelinus fontinalis | M22710-11 | Wild salmon |
| GCF_001902025.1 | Canada | Salvelinus fontinalis | m23067-09 | Wild salmon |
| GCF_900445115.1 | Unknown | Salmo salar | NCTC12959 | Reared salmon |
| GCF_002180335.1 | Poland | Unknown | O23A | Environmental |
| GCA_029159705.1 | USA | Oncorhynchus tshawytscha | OTH-19-VL-OH-WA-0027 | Reared salmon |
| GCA_029158765.1 | USA | Oncorhynchus tshawytscha | OTH-19-VL-OH-WA-0045 | Reared salmon |
| GCA_029153915.1 | USA | Oncorhynchus mykiss | OTH-20-OH-WA-0033 | Reared salmon |
| GCA_029153895.1 | USA | Oncorhynchus kisutch | OTH-20-OH-WA-0050 | Reared salmon |
| GCA_038847985.1 | USA | Anoplopoma fimbria | OTH-20-OH-WA-0059 | Other fish |
| GCA_029153855.1 | USA | Oncorhynchus mykiss | OTH-20-OH-WA-0076 | Reared salmon |
| GCA_029153875.1 | USA | Oncorhynchus tshawytscha | OTH-20-OH-WA-0077 | Reared salmon |
| GCA_039659795.1 | USA | Oncorhynchus mykiss | OTH-21-VL-OH-WA-0017 | Reared salmon |
| GCA_039659575.1 | USA | Oncorhynchus tshawytscha | OTH-21-VL-OH-WA-0049 | Reared salmon |
| GCA_039659495.1 | USA | Oncorhynchus kisutch | OTH-21-VL-OH-WA-0050 | Reared salmon |
| GCA_039659555.1 | USA | Oncorhynchus tshawytscha | OTH-21-VL-OH-WA-0070 | Reared salmon |
| GCA_039659595.1 | USA | Oncorhynchus kisutch | OTH-21-VL-OH-WA-0071 | Reared salmon |
| GCA_039659635.1 | USA | Oncorhynchus kisutch | OTH-21-VL-OH-WA-0091 | Reared salmon |
| GCA_039659695.1 | USA | Oncorhynchus kisutch | OTH-21-VL-OH-WA-0092 | Reared salmon |
| GCA_032495985.1 | Canada | Salmo salar | OTH-21-VL-ON-BC-0001 | Reared salmon |
| GCA_032496025.1 | Canada | Anoplopoma fimbria | OTH-21-VL-ON-BC-0003 | Other fish |
| GCA_033145875.1 | Canada | Salmo salar | OTH-21-VL-ON-BC-0004 | Reared salmon |
| GCA_032496085.1 | Canada | Anoplopoma fimbria | OTH-21-VL-ON-BC-0005 | Other fish |
| GCA_032496165.1 | Canada | Salmo salar | OTH-21-VL-ON-BC-0008 | Reared salmon |
| GCA_032496445.1 | Canada | Salmo salar | OTH-21-VL-ON-BC-0009 | Reared salmon |
| GCA_032496465.1 | Canada | Salmo salar | OTH-21-VL-ON-BC-0012 | Reared salmon |
|  | Chile | Salmo salar | PM-118328 | Reared salmon |
|  | Chile | Salmo salar | PM-118565 | Reared salmon |
|  | Chile | Salmo salar | PM-119448 | Reared salmon |
| GCF_002313065.1 | South Korea | Sebastes schlegelii | RFAS1 | Other fish |
| GCA_001499805.2 | France | Fish | RS534 | Reared salmon |
| GCA_019443805.1 | China | Turbot | RZ6S-1 | Other fish |
| GCA_002214245.1 | China | Salmo salar | S121 | Reared salmon |
| GCF_002214305.1 | China | Salmo salar | S44 | Reared salmon |
| GCF_002214265.1 | China | Salmo salar | S68 | Reared salmon |
| GCA_008370735.1 | Canada | Salvelinus fontinalis | SHY16-3432 | Wild salmon |
| GCA_012931585.1 | China | Unknown | SRW-OG1 | Other fish |
| GCA_029906615.1 | USA | Anoplopoma fimbria | T30 | Other fish |
| GCA_023108375.1 | Norway | Atlantic salmon | VI-88/09/03175 | Reared salmon |
| GCA_001466435.1 | India | Fish | Y567 | Other fish |
| GCF_001481545.2 | India | Fish | Y577 | Other fish |
| GCF_002093675.1 | China | Coreius guichenoti | YK | Other fish |

Table S3. Results of PCR assay targeting a gene of the capsule biosynthesis cluster present in *vapA*-absent variants (in bold).

| ID | *vapA* | *fstA* | *cap* |
| --- | --- | --- | --- |
| AY-2485 | p | p | n |
| 2 | p | p | n |
| 3 | p | p | n |
| 4 | p | p | n |
| 5 | p | p | n |
| 6 | p | p | n |
| 7 | p | p | n |
| 8 | p | p | n |
| 9 | p | p | n |
| 10 | p | p | n |
| 11 | p | p | n |
| 12 | p | p | n |
| 13 | p | p | n |
| 14 | p | p | n |
| 15 | p | p | n |
| 16 | p | p | n |
| 17 | p | p | n |
| 18 | p | p | n |
| 19 | p | p | n |
| 20 | p | p | n |
| 21 | p | p | n |
| 22 | p | p | n |
| 23 | p | p | n |
| 24 | p | p | n |
| 25 | p | p | n |
| PM-118328 | p | p | n |
| 27 | p | p | n |
| PM-118565 | **n** | p | **p** |
| 29 | **n** | p | **p** |
| PM-119448 | **n** | p | **p** |
| 31 | **n** | p | **p** |
| 32 | p | p | n |
| 33 | p | p | n |
| 34 | p | p | n |
| 35 | p | p | n |
| 36 | p | p | n |
| 37 | p | p | n |
| 38 | p | p | n |
| 39 | p | p | n |
| 40 | p | p | n |
| 41 | p | p | n |
| 42 | **n** | p | **p** |
| 43 | **n** | p | **p** |
| 44 | **n** | p | **p** |
| 45 | p | p | n |
| 46 | p | p | n |
| 47 | **n** | p | **p** |

*cap*: capsular polysaccharide biosynthesis protein. Primer used: Fwd- TGCGCCATGGCTATTCTTCT; Rev-TGAGGTCAGAAATGATAGTGGCA; chemistry, SYBR Green.

Table S4. Annotation of *vapA* locus found in SRW-OG1 genome (from left to right Figure 4).

1. HED66_RS02075 : UDP-N-acetylglucosamine 4,6-dehydratase
2. HED66_RS02080 : LegC family aminotransferase
3. HED66_RS02085 : MaoC family dehydratase
4. HED66_RS02090 : GNAT family N-acetyltransferase
5. HED66_RS02095 : UDP-N-acetylglucosamine 2-epimerase
6. HED66_RS02100 : PIG-L deacetylase family protein
7. HED66_RS02105 : N-acetylneuraminate synthase
8. HED66_RS02110 : acetyltransferase
9. HED66_RS02115 : nucleotidyltransferase family protein
10. HED66_RS02120 : acylneuraminate cytidylyltransferase family protein
11. HED66_RS02125 : polysialyltransferase family glycosyltransferase
12. HED66_RS02130 : oligosaccharide flippase family protein
13. HED66_RS02135 : EpsG family protein
14. HED66_RS02140 : glycosyltransferase
15. HED66_RS02145 : FAD-dependent oxidoreductase
16. HED66_RS02150 : glycosyltransferase
17. HED66_RS02155 : sugar transferase
18. HED66_RS21300 : nucleotidyltransferase

Figure S1. Growth kinetics of *vapA+* Chilean isolates in TSB. Experiment was conducted using conditions inferred from Figure 2A: temperature range 10 °C > t < 23°C and mild saline conditions (0-0.5%).


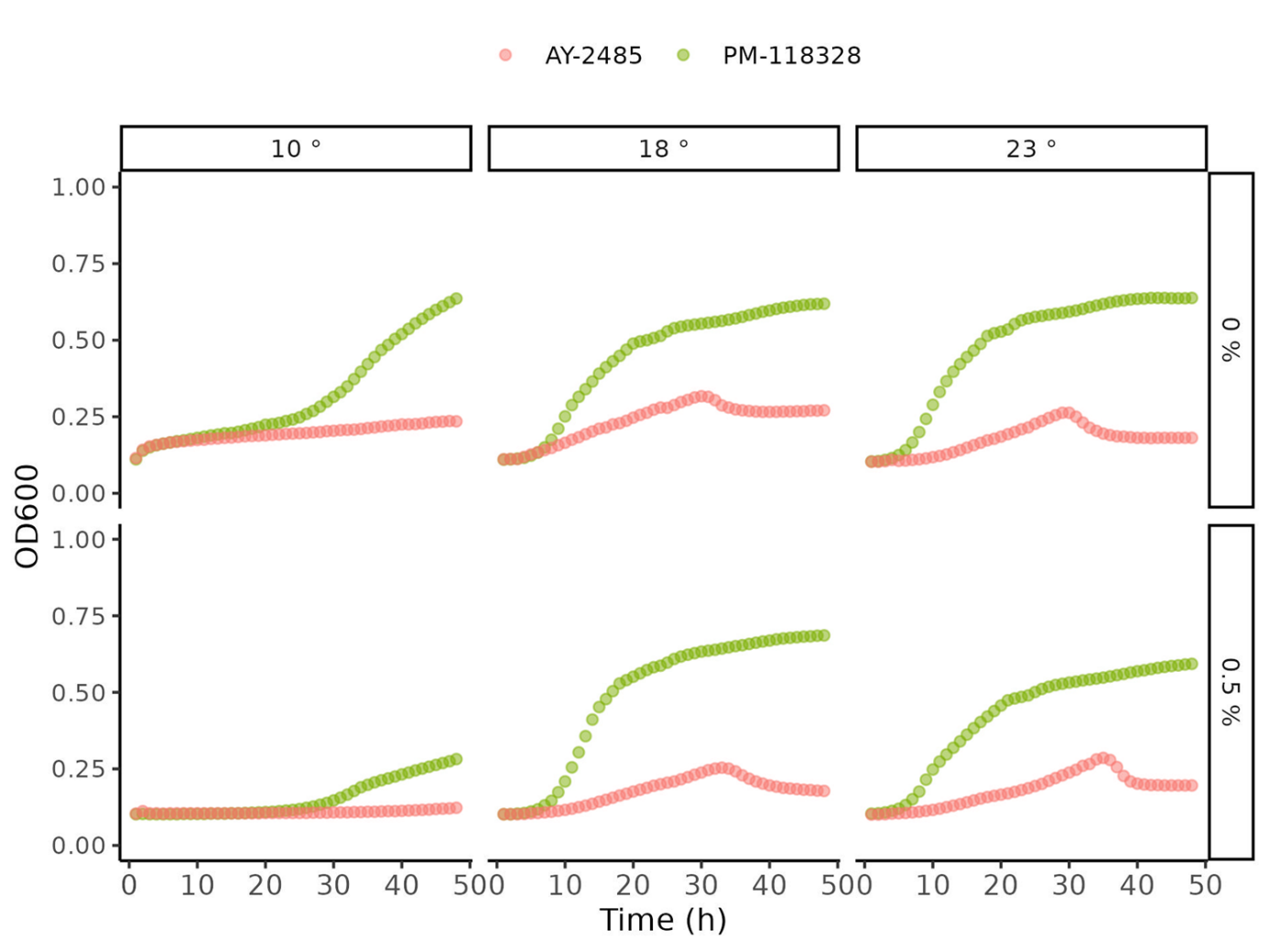


Figure S2. LD50 calculations for different *A. salmonicida* strains (Figure 6). Statistical significance is shown below (Bonferroni’s correction, Probit R Package).


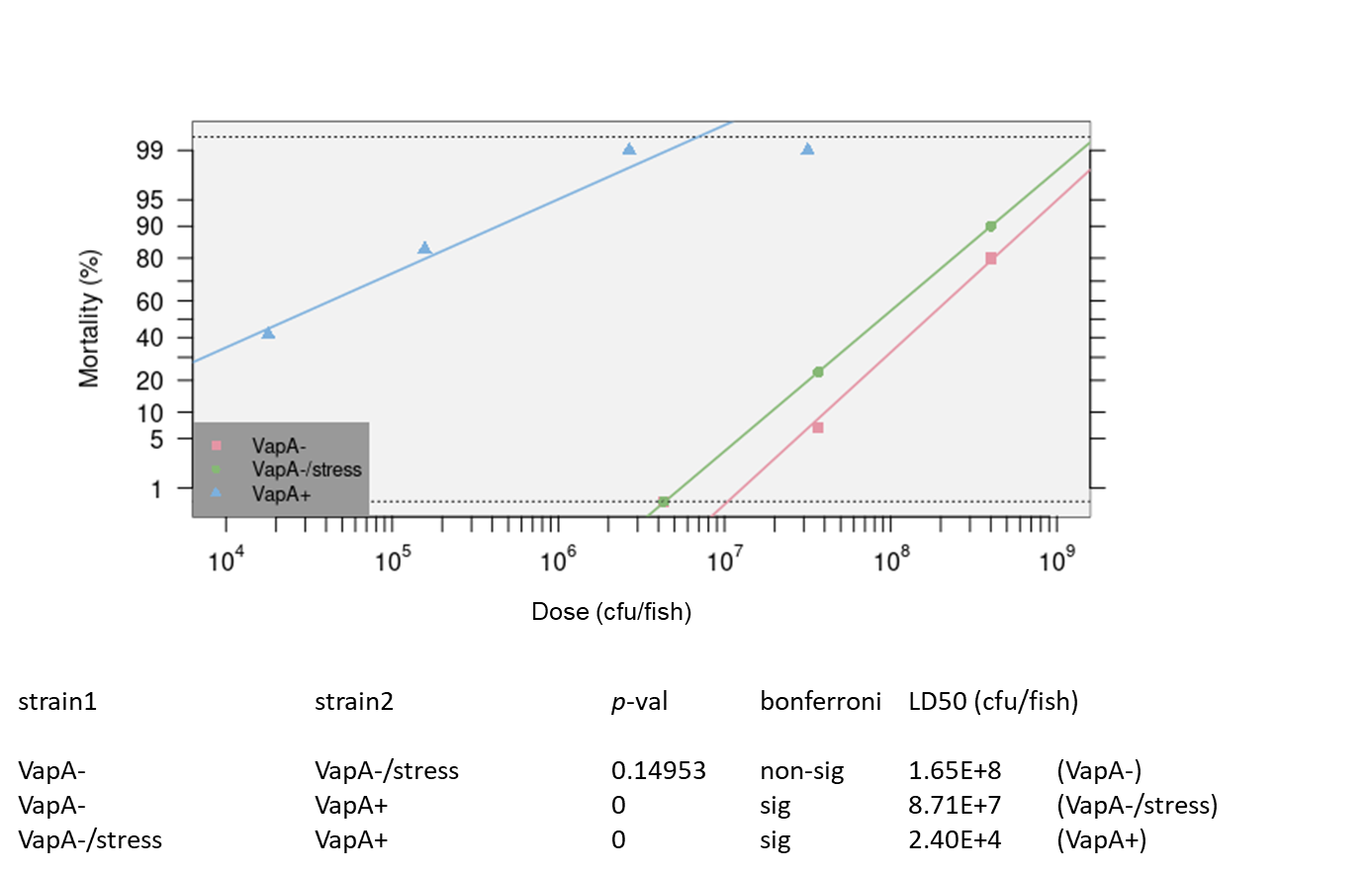

Supplement: Supplementary file 1 [file DataSheet1.docx]
